# Evolution of a globally unique SARS-CoV-2 Spike E484T monoclonal antibody escape mutation in a persistently infected, immunocompromised individual

**DOI:** 10.1093/ve/veac104

**Published:** 2022-11-05

**Authors:** Peter J Halfmann, Nicholas R Minor, Luis A Haddock III, Robert Maddox, Gage K Moreno, Katarina M Braun, David A Baker, Kasen K Riemersa, Ankur Prasad, Kirsten J Alman, Matthew C Lambert, Kelsey Florek, Allen Bateman, Ryan Westergaard, Nasia Safdar, David R Andes, Yoshihiro Kawaoka, Madiha Fida, Joseph D Yao, Thomas C Friedrich, David H O’Connor

**Affiliations:** Department of Pathobiological Sciences, University of Wisconsin-Madison, 2015 Linden Dr, Madison, WI 53706, USA; Department of Pathology and Laboratory Medicine, 3170 UW Medical Foundation Centennial Building (MFCB), 1685 Highland Avenue, Madison, WI 53705, USA; Department of Pathobiological Sciences, University of Wisconsin-Madison, 2015 Linden Dr, Madison, WI 53706, USA; Department of Pathology and Laboratory Medicine, 3170 UW Medical Foundation Centennial Building (MFCB), 1685 Highland Avenue, Madison, WI 53705, USA; Department of Pathology and Laboratory Medicine, 3170 UW Medical Foundation Centennial Building (MFCB), 1685 Highland Avenue, Madison, WI 53705, USA; Department of Pathobiological Sciences, University of Wisconsin-Madison, 2015 Linden Dr, Madison, WI 53706, USA; Department of Pathology and Laboratory Medicine, 3170 UW Medical Foundation Centennial Building (MFCB), 1685 Highland Avenue, Madison, WI 53705, USA; Department of Pathobiological Sciences, University of Wisconsin-Madison, 2015 Linden Dr, Madison, WI 53706, USA; Division of Allergy, Pulmonary and Critical Care Medicine, School of Medicine and Public Health, 1685 Highland Avenue, 5158 Medical Foundation Centennial Building, Madison, WI 53705-2281, USA; University of Wisconsin Division of Infectious Disease, Room 5275-07C, 1685 Highland Avenue, Madison, WI 53705, USA; University of Wisconsin Division of Infectious Disease, Room 5275-07C, 1685 Highland Avenue, Madison, WI 53705, USA; Wisconsin State Laboratory of Hygiene, 2601 Agriculture Drive, PO Box 7996, Madison, WI 53707, USA; Wisconsin State Laboratory of Hygiene, 2601 Agriculture Drive, PO Box 7996, Madison, WI 53707, USA; Department of Medicine, 1685 Highland Avenue, 5158 Medical Foundation Centennial Building, Madison, WI 53705, USA; Department of Medicine, 1685 Highland Avenue, 5158 Medical Foundation Centennial Building, Madison, WI 53705, USA; Department of Medicine, 1685 Highland Avenue, 5158 Medical Foundation Centennial Building, Madison, WI 53705, USA; Department of Pathobiological Sciences, University of Wisconsin-Madison, 2015 Linden Dr, Madison, WI 53706, USA; Division of Infectious Diseases, Mayo Clinic, 200 First St. SW, Rochester, Rochester, Minnesota 55905, USA; Department of Laboratory Medicine and Pathology, Mayo Clinic, 200 First St. SW, Rochester, MN 55905, USA; Department of Pathobiological Sciences, University of Wisconsin-Madison, 2015 Linden Dr, Madison, WI 53706, USA; Department of Pathology and Laboratory Medicine, 3170 UW Medical Foundation Centennial Building (MFCB), 1685 Highland Avenue, Madison, WI 53705, USA

**Keywords:** SARS-CoV-2, prolonged infection, antibody escape, immunocompromised host, novel mutation, Spike protein

## Abstract

Prolonged infections in immunocompromised individuals may be a source for novel Severe Acute Respiratory Syndrome Coronavirus 2 (SARS-CoV-2) variants, particularly when both the immune system and antiviral therapy fail to clear the infection and enable within-host evolution. Here we describe a 486-day case of SARS-CoV-2 infection in an immunocompromised individual. Following monotherapy with the monoclonal antibody Bamlanivimab, the individual’s virus acquired resistance, likely via the earliest known occurrence of Spike amino acid variant E484T. Recently, E484T has arisen again as a derivative of E484A in the Omicron Variant of Concern, supporting the hypothesis that prolonged infections can give rise to novel variants long before they become prevalent in the human population.

## Introduction

The host immune response typically clears SARS-CoV-2 infections by 15–25 days (Fang et al. 2020; Noh et al. 2020; Zhou et al. 2020; Simmonds, Williams, and Harvala 2021), and this short duration of active virus replication limits within-host evolution. In contrast, infections in immunocompromised individuals can be prolonged, with the longest previously documented infection lasting 471 days (Chaguza et al. 2022). Prolonged infections may facilitate SARS-CoV-2 evolution by providing time for with-in-host selection to act, potentially driving the emergence of immunologic escape variants (reviewed in (Harvey et al. 2021)).

Spike residue E484 lies in the receptor-binding domain, which is the target for approximately 90 per cent of neutralizing antibodies (Tortorici et al. 2020). A recent study exposed a VSV-eGFP-SARS-CoV-2-S chimeric virus to nineteen neutralizing monoclonal antibodies (mAbs) and found that substitutions appeared at E484 more frequently than at any other residue, suggesting that this residue plays a key role in antibody escape. E484A, E484D, E484G, and E484K all reduce the neutralization activity of antibodies in convalescent plasma (Liu et al. 2021). Spike E484K is the best studied of these variants and is particularly impactful for the mAb Bamlanivimab, reducing susceptibility by more than 2,360-fold (Pica and Carter 2021). This prompted the US Food and Drug Administration to revoke Emergency Use Authorization for Bamlanivimab on 16 April 2021 (Pica and Carter 2021).

Another variant at this residue, Spike E484A, is found in the highly transmissible Omicron (B.1.1.529) Variant of Concern, which may have emerged from a prolonged infection in an immunocompromised individual (Cele et al. 2021). Providing further support for this hypothesis, E484A variants have previously arisen in immunocompromised individuals with prolonged infection (Choi et al. 2020; Cele et al. 2021). SARS-CoV-2 culture in the presence of mAbs can also select E484A *in vitro* (Ku et al. 2021). E484A was transiently detected in one patient receiving Bamlanivimab monotherapy, but the impact of this mutation on Bamlanivimab recognition was not directly tested (Peiffer-Smadja et al. 2021).

Here, we report a 16 month-long SARS-CoV-2 infection in an immunocompromised individual. Following unsuccessful Bamlanivimab monotherapy, many substitutions were detected in the consensus sequence of the infecting virus, including one encoding Spike E484T, a substitution that had not been reported in public SARS-CoV-2 sequence databases prior to this infection. Virus isolated from this patient encoding Spike E484T was highly resistant to Bamlanivimab, but remained susceptible to three other commercial mAbs. These findings highlight the necessity of frequent testing, viral sequencing and proactive treatment for prolonged SARS-CoV-2 infections occurring in immunocompromised individuals.

## Results

### Case background

The patient described in this case is in their 50s and suffers from a combination of primary immunodeficiency disorders and other comorbidities. These include common variable immunodeficiency with recurrent lower respiratory tract infections, Evans syndrome and associated autoimmune cytopenias, and extranodal mucosa-associated lymphoid tissue lymphoma of the lung. To manage these disorders, the patient has received monthly intravenous immunoglobulin infusions since 2015. Notably, the patient also received 700 mg of intravenous Bamlanivimab mAb therapy 198 days after their diagnosis with COVID-19. Later in their infection, they also received 3 days of outpatient VaxPlasma, a high-titer donor blood plasma from vaccinated individuals. The patient eventually received a negative Polymerase Chain Reaction (PCR) result at the 525th day post-diagnosis, with two additional negative results after 8 months.

Note that the above case history has been abbreviated; the full case description can be found in the Supplemental Materials (see Unabbreviated Case Description).

### Detection and sequencing of SARS-CoV-2 over 16 months

**Figure 1.**
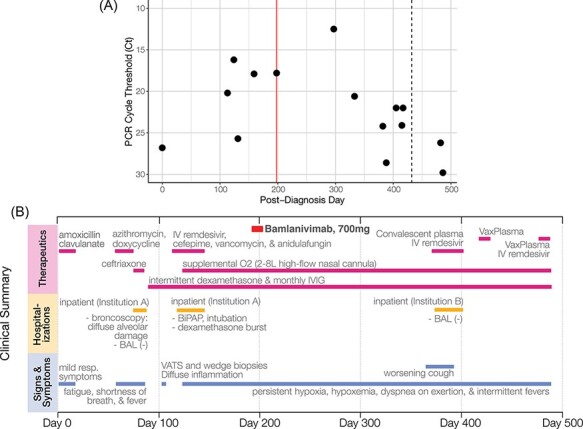
(A) RT-qPCR cycle threshold values for patient nasopharyngeal swab specimens collected over more than 400 days of follow-up. The vertical line marks Post-diagnosis Day 198, when the patient received the Bamlanivimab intravenous mAb treatment (700 mg). The dotted line on Post-diagnosis Day 432 indicates a positive PCR test but no available Ct value. Note that Ct values are inverted to correspond with the inverse relationship between PCR Ct threshold value and nasal RNA copy number. (B) Timeline of clinically relevant information for the patient’s chronic infection, including symptoms, therapeutics, and hospitalizations.

**Figure 2.**
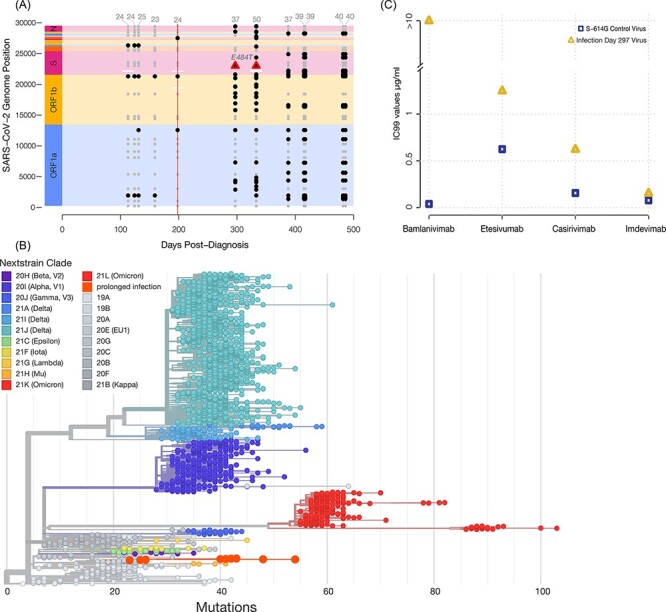
(A) Consensus-level mutations (present in >50% of reads) across the SARS-CoV-2 genome throughout infection of the immunocompromised individual. Gray points represent consensus-level mutations that persisted throughout all sampled time points and may have been inherited from the infecting lineage. Black points represent consensus-level mutations that emerged or disappeared during the chronic infection. The vertical red line at Day 198 represents Bamlanivimab intravenous mAb treatment (700 mg). Amplicon dropouts are depicted as white horizontal bars. Consensus-level E484T mutations in Spike are shown as triangles. (B) Globally subsampled SARS-CoV-2 phylogeny with chronically infected patient’s twelve virus sequences. Patient sequences are indicated with enlarged dots toward the bottom of the tree. The patient’s virus descends from an ancestor in the lineage B.1.2. The phylogeny was constructed using UShER (https://genome.ucsc.edu/cgi-bin/hgPhyloPlace), a tool that allows rapid placement of sequences on the SARS-CoV-2 phylogeny. (C) The concentration of four commercially available antibody treatments required to neutralize 99% of the chronically infected patient’s SARS-CoV-2. While Etesevimab (Eli Lilly), Casirivimab (Regeneron), and Imdevimab (Regeneron) were able to neutralize virus near or below 1 μg/ml, Bamlanivimab was unable to neutralize the virus at concentrations as high as 10 μg/ml, indicating antibody escape after the previous Bamlanivimab treatment.

Reverse Transcription Quantitative Polymerase Chain Reaction (RT-qPCR) Ct values were below twenty-seven in the majority of nasopharyngeal swab PCR tests (Fig. 1A). Additionally, viral isolates from the immunocompromised individual were sequenced at twelve time points, beginning on Post-diagnosis Day 113 (Supplementary Table S1). We found that consensus sequences from each of these time points are more closely related to one another than other sequences in public repositories. Furthermore, all twelve sequences classify as Pango lineage B.1.2, a lineage that peaked in late 2020 at roughly 20 per cent of global sequenced cases but was seldom detected throughout 2021, save for this patient’s infection. B.1.2 was implicated in the first known SARS-CoV-2 reinfection case and has also infected white-tailed deer and snow leopards (Fonseca et al. 2021; Gangavarapu et al. 2022; Hale et al. 2022; Kuchipudi et al. 2022; Tsueng et al. 2022; Wang et al. 2022). The clustering of all twelve sequences on a single long branch (Fig. 2B), together with their classification as B.1.2 even long after that lineage was prevalent, is most consistent with a prolonged infection rather than reinfection. However, a household member tested positive for SARS-CoV-2 on Day 87, and it is possible that the immunocompromised individual cleared their infection after the previously confirmed PCR test on Day 0, but was reinfected by their household contact. Unfortunately, no samples from this household member are available to investigate this possibility.

Sequencing revealed a cumulative sixty-two consensus-level mutations from the Wuhan-1 reference (GenBank MN908947.3), some of which persisted for the duration of the infection and some that appeared and later disappeared. At the earliest time point for which sequencing data are available, taken 113 days after the initial diagnosis, we detected twenty-four mutations that separated the patient’s virus from Wuhan-1, nineteen of which were nonsynonymous (see Supplementary Table S3). This number of mutations roughly matches the number of mutations across B.1.2 samples, which typically range from 17 to 24. Several of the nonsynonymous mutations in this infection were prevalent when the infection presumably began, including ORF1ab T265I, found in the Beta VOC, which was often detected in mid-to-late 2020 (Laha et al. 2020; Alai et al. 2021; Slavov et al. 2021); ORF1ab P4715L, which arose with Spike D614G by mid-2020 (Fang et al. 2021); and Spike D614G, which became one of the most prevalent variants by mid-2020 and was shared by the persistently infecting virus’s ancestral lineage, B.1.2 (Gangavarapu et al. 2022; Tsueng et al. 2022). The virus also showed the then-prevalent Spike Q677H, which arose in at least seven SARS-CoV-2 lineages independently by early 2021 (Ford et al. 2021; Pater et al. 2021; Tomkins-Tinch et al. 2021); ORF3a A23V, which became globally prevalent among B.1.1.7 sequences by mid-2021 (Arantes et al. 2021; Benslimane et al. 2021); and Nucleocapsid variants N P67S, N P199L, and N D377Y (Ford et al. 2021; Hodcroft et al. 2021; Pater et al. 2021; Tu et al. 2021; Zimerman et al. 2022).

We did not assess insertions and deletions in our Oxford Nanopore Technologies (ONT) sequence data. However, in our Illumina sequence data, we found no strong evidence of insertions, although we did find strong evidence for two unique deletions, both in Spike. On Day 159, we saw a low-frequency (0.31), in-frame deletion of thirty-nine nucleotides at Position 21,747, where a deletion has occurred in only twenty-six International Nucleotide Sequence Database Collaboration (INSDC) accessioned sequences, according to Open cov-SPECTRUM (Chen et al. 2021a). Starting on Day 124 and continuing for the rest of our time points, we also observed an in-frame deletion of twelve nucleotides at Position 21,981, where deletions have occurred in 0.01 per cent of INSDC sequences (see Supplementary Table S4).

In addition to these deletions, we detected consensus-level single-nucleotide substitutions that were not globally prevalent at the time of infection. These included ORF1ab V1056L, a mutation in 0.11 per cent of INSDC-accessioned sequences, as of September 2022, that may be involved in polyprotein processing (Chan et al. 2021); ORF1ab L3606F, a mutation at 1.69 per cent global prevalence that may be associated with low pathogenicity (Koyama and Parida 2020; Ghanchi et al. 2020; Toyoshima et al. 2020); Spike T1117I, which has been detected in 4849 INSDC sequences, emerged late in the infection on Day 388 (Molina-Mora 2022); N P383L, another rare mutation in 0.15 per cent of INSDC sequences, is nonetheless mentioned in a case of reinfection (Camargo et al. 2021). E T30I, one of the most frequent mutations in immunocompromised individuals (Borges et al. 2021; Chaudhry et al. 2022; Wilkinson et al. 2022), also arose during this individual’s infection, although we no longer detected this mutation from Infection Day 159 forward. Finally, Spike T19I, which is also now found in the Omicron BA.2 sublineage, arose after treatment with Bamlanivimab. A number of studies dating to 2022 associate this mutation with reduced Spike-protein-mediated infectivity (Chan et al. 2022; Pastorio et al. 2022; Rahimi and Abadi 2022; Rajpal et al. 2022).

In addition to Spike T19I, we detected a second Spike mutation that is now found in Omicron-lineage viruses: Spike E484A, caused by a point mutation at Nucleotide Position 23,013, GAA > G**C**A, which is found in the original Omicron BA.1 lineage and its descendants. Like Envelope Codon 30, Spike Codon 484 shows some of the most frequently recurring mutations among published prolonged infection cases (Wilkinson et al. 2022) and is part of the particularly exposed E6 epitope identified by Sikora et al. (2021) (see Figure 5 in that study). Spike E484A persisted in the virus consensus sequence for five further time points, at least through Post-diagnosis Day 198. On that day, the patient received the Bamlanivimab mAb therapy, and their virus was subsequently not sequenced for the following 99 days. There was no additional testing or sequencing during this interval, one of the limitations of this study.

### Emergence of the globally unique Spike E484T and other mutations after Bamlanivimab treatment

On Post-diagnosis Day 297, our first sequencing time point after Bamlanivimab treatment, we observed thirty-seven consensus mutations from the Wuhan-1 sequence (Fig. 2A), twenty-five of which were not also detected in the consensus sequences at previous time points. One of these mutations, ORF1ab V4102I, was a low-frequency within-host variant at Infection Day 159. Another mutation that arose at Day 297 was an additional mutation in Codon 848, this time at Nucleotide Position 23,012, G**C**A > **AC**A. On its own, this G-to-A nucleotide substitution would cause Spike E484K, a mutation with known antibody escape properties (Ferrareze et al. 2021; Jangra et al. 2021; Liu et al. 2021; Nonaka et al. 2021; Pica and Carter 2021). However, together with the A-to-C substitution that caused E484A, the newer substitution changed the Codon 484 amino acid residue to threonine (T). As of September 2022, this is the earliest documented occurrence of Spike E484T and the only occurrence in 2021. Since then, Spike E484T has been documented in thirty-five additional INSDC sequences, all on an Omicron genomic background. More than 5 months passed between the detection of Spike E484T in this infection and its detection in Omicron samples.

Spike E484T remained at high frequency (0.99) in our sequencing reads in our next sequencing time point, Post-diagnosis Day 333. However, by the following time point on Day 388, the nucleotide mutation that caused E484T was absent in our consensus sequence, with a frequency that was too low in our Illumina reads to differentiate it from a sequencing error. Without the nucleotide substitution causing E484T, Spike Codon 484 returned to E484A in 99 per cent of sequencing reads, which was its frequency for the remainder of our sequencing time points (see Supplementary Fig. S1 and Supplementary Table S4). While we cannot rule out that the substitution causing E484T and other mutations persisted at very low frequency, this outcome is indistinguishable from a sequencing error.

After Post-diagnosis Day 333, we detected additional thirteen consensus-level mutations, eleven of which were nonsynonymous, and by our final time point, there were forty mutations separating our consensus sequence from Wuhan-1. In contrast with some prolonged infection studies showing a greater number of within-host mutations than expected given the global SARS-CoV-2 substitution rate (e.g. Chaguza et al. 2022) this accumulation of mutations is roughly in line with the amount expected for viruses isolated on these dates (see Supplementary Fig. S2), although it is important to note that substitution rates cannot be inferred from consensus sequences.

### Culture and neutralization assay of virus exhibiting Spike E484T

We next examined whether the virus harboring E484T could escape mAb recognition. We cultured autologous virus in Vero E6/TMPRSS2 cells from Infection Day 297, when we first detected twenty-five new consensus mutations including Spike E484T (Fig. 2A), and performed a neutralization assay with four commercially available antibodies (Fig. 2C). Eli Lilly’s Etesevimab, as well as Regeneron’s Casirivimab and Imdevimab, neutralized the Post-diagnosis Day 297 virus at relatively similar concentrations. Overall, 99 per cent of cell death was prevented with 1.25 µg/ml of Etesevimab, 0.62 µg/ml of Casirivimab, and 0.16 µg/ml of Imdevimab. However, Eli Lilly’s Bamlanivimab could not neutralize the virus at concentrations as high as 10 µg/ml, indicating that the virus isolated from the patient after Bamlanivimab treatment is no longer susceptible to this antibody. This virus expressed E484T among three other Spike amino acid substitutions (see Supplementary Table S2). We cannot determine whether E484T alone confers effective escape from Bamlanivimab, but we note that E484T is the only site among the substitutions in this virus isolate that affects Spike sites with known antigenic impact.

## Discussion

The degree of genetic divergence we document in this patient’s infection highlights the potential role of immunocompromised individuals in generating novel variants. We also describe the earliest case of the Spike E484T variant, which arose after treatment with the Bamlanivimab monotherapy. This emergence of novel variants after immunocompromised patients receive Bamlanivimab is by now a well-documented pattern (Jensen et al. 2021; Peiffer-Smadja et al. 2021; Truffot et al. 2021; Chaguza et al. 2022; Vellas et al. 2022), although never with the substitution Spike E484T. While E484T appears to be novel for SARS-CoV-2, the bat betacoronavirus RaTG13, one of the coronaviruses most similar to SARS-CoV-2 in terms of nucleotide identity, also bears a threonine at Spike Codon 484.

While knowledge of the functional impact of E484T is limited, *in silico* E484T mutants of the SARS-CoV-2 Spike protein showed increased binding of mouse angiotensin converting enzyme-2 (ACE2) receptors, the key for viral entry into the cell, by more than 16-fold (Li et al. 2021).

Two common limitations in prolonged infection studies, including this one, are (1) the lack of residual specimens for retrospective analysis and (2) the collection of specimens from only a single tissue reservoir (in most cases, the nasopharynx). Given the volume of specimen required for RT-qPCR, most providers discard residual nasopharyngeal swab specimens after testing, and few providers collect specimens other than nasopharyngeal swabs, e.g. stool or serum samples. However, this case highlights the potential value of archiving test-positive specimens representing multiple tissue reservoirs in immunocompromised, chronically infected individuals. If residual samples were archived in this case, it would be possible to determine conclusively whether the E484A variant arose *de novo* in this individual or was present in the infecting lineage. Similar studies of prolonged SARS-CoV-2 infections in immunocompromised individuals (Avanzato et al. 2020; Choi et al. 2020; Kemp et al. 2021; Weigang et al. 2021), the lack of Spike E484A in the ancestral B.1.2 lineage, and the lack of other E484A sequences in Wisconsin during late 2020 all indicate that this variant likely arose *de novo* in the months following the initial infection, but this cannot be confirmed without residual samples. Similarly, because only nasopharyngeal swabs were taken, it is unknown if the mutations we document here represent only that fraction of within-host genetic diversity that is present in the nasopharynx. Over the course of a prolonged infection, highly divergent viruses may evolve in other tissue reservoirs that are not sampled with nasopharyngeal swabs. As such, it may be necessary to collect nasopharyngeal samples alongside other sample types to capture the full breadth of viral genetic diversity and evolution in prolonged infections. To solve these limitations to the study of prolonged infections, a national or global registry to collect all available sample types from individuals with prolonged infection and to study these individuals in greater numbers may be warranted.

Although SARS-CoV-2 sequence data are limited in the county where this individual lives, surveillance sequencing suggests that there was no onward spread of the individual’s virus, notwithstanding the household case of unknown provenance as well as undetected superinfections in other individuals.

This lack of spread is particularly consequential for the Bamlanivimab-evading day-297 virus, which bore Spike E484T along with cumulative thirty-six other mutations. That E484T was unique to this individual raises the possibility that E484T, and the other mutations that emerged with it (Supplementary Fig. S1), may be competitive in the presence of Bamlanivimab but incur a fitness cost in its absence. However, this hypothesis would require that the monoclonal persisted much longer in the immunocompromised patient’s serum than Bamlanivimab’s previously documented half-life of 17 days (Chen et al. 2021b; Chigutsa et al. 2021). Like Spike E484T, the functional and fitness impact of most of the sixty-two cumulative consensus-level mutations is as-yet unknown. Only Spike D614G, a characteristic mutation in the ancestral B.1.2 lineage, is known to increase transmissibility between hosts. It is important to note, although, that increased transmissibility need not evolve in all, or even most, prolonged infections in order for some such infections to contribute to the emergence of novel variants. We expect that the trajectories of within-host evolution differ among different prolonged infections, and more study is needed to identify situations that might particularly favor the emergence of transmissible variant viruses from such individuals.

Another, non-mutually exclusive explanation for the lack of onward spread is that the patient’s collaboration with state and local public health officials has been successful at managing their infection, especially after Spike E484T was detected. Indeed, it is to their credit that this case report involves only one individual. While this is only one case, it foreshadows the difficult ethical questions that come up in any case of prolonged infection in an immunocompromised individual, of which there are millions in the USA (Wallace et al. 2021). These questions echo previous controversies, such as the isolation of individuals with highly drug-resistant tuberculosis (Parmet 2007), but may be amplified in the USA by the high incidence of SARS-CoV-2 and the large number of immunosuppressed/immunocompromised individuals.

Finally, it is worth considering whether individuals with prolonged SARS-CoV-2 shedding can presage the course of future virus evolution. The E484T variant was identified in this infection more than 5 months before it occurred in other samples. Similarly, E484A was identified in multiple immunosuppressed individuals before it appeared as a signature mutation in the Omicron Variant of Concern.

Surveillance programs that concentrate on identifying prolonged infections and characterizing viral evolution in such cases may, therefore, provide important insights into the potential future directions of SARS-CoV-2 evolution.

### Study limitations

In this study, we use sequence data to document the evolution of a SARS-CoV-2 population sampled from the nasopharynx of a single host. As such, a number of limitations apply to our results. Perhaps most importantly, the patterns of SARS-CoV-2 evolution in a single immunocompromised host cannot be used to forecast the emergence of specific future variants. Our study also works around notable gaps in sequence data availability; the first 112 days of infection went without sequencing, as did the 99 days following Bamlanivimab treatment while the patient’s symptoms largely subsided. The gap at the beginning of the infection makes it impossible to ascertain the origin of the virus and also limits our ability to time the emergence of many mutations, as discussed above. We are also unable to make any claims about the viability or infectivity of the virus, save for Infection Day 297, as autologous virus was not cultured at any other time points. We also did not isolate the impact of E484T in an isogenic background. Other samples, including serum samples, are no longer available to assess the titers of specific antibodies. Finally, we are unable to assess the transmission of this individual’s virus because of the paucity of sequence data from their household and surrounding community. These limitations all contribute to our call for more frequent archiving of test-positive specimens from immunocompromised individuals, especially those coming from prolonged infections, as these samples are invaluable for retrospective sequence analysis.

## Materials and methods

### Ethics statement

Collection and testing of biological specimens and protected health information were performed in concordance with the University of Wisconsin Insitutional Review Board # 2021-0076 following informed consent from the patient.

### SARS-CoV-2 quantification

Diagnostic RT-qPCR was performed on nasopharyngeal swab specimens at Exact Sciences (Madison, WI), University of Wisconsin Hospitals and Clinics, or the Mayo Clinic for all time points except Day 297. On Day 297, the patient was tested with a qualitative Panther Fusion assay from Hologic, Inc. (Marlborough, MA) for clinical care purposes (Supplementary Table S1).

To obtain a semi-quantitative measurement on Day 297, we isolated viral RNA from an additional nasopharyngeal swab sample using the Total Nucleic Acid kit for the Maxwell RSC instrument (Promega, Madison, WI). We used the N1 qRT-PCR assay developed by the United States Centers for Disease Control and Prevention and commercially available from Integrated DNA Technologies (IDT) (Coralville, IA). The assay was run on a LightCycler 96 instrument (Roche, Indianapolis, IN) with the Taqman Fast Virus 1-Step Master Mix enzyme (Thermo Fisher, Waltham, MA).

### SARS-CoV-2 culture

Vero E6/TMPRSS2 cells obtained from the Japanese Collection of Research Bioresources Cell Bank (1819) were grown in Dulbecco’s Modified Eagle Medium supplemented with 5 per cent fetal calf serum, HEPES buffer, amphotericin B, and gentamicin sulfate. The immunocompromised individual’s virus was isolated from a Day 297 nasopharyngeal swab sample cultured on Vero E6/TMPRSS2 cells. We compared the patient’s virus with a prototypical 2020 SARS-CoV-2 control virus whose Spike protein sequence matched the ancestral Wuhan-1 reference except for D614G (SARS-CoV-2/UT-HP095-1N/Human/2020/Tokyo), which has been described previously (Imai et al. 2020, 2021).

### Monoclonal antibody susceptibility assay

mAbs derived from the sequences of commercial therapeutic antibodies (Regeneron and Eli Lilly) were purchased from InvivoGen (Casirivimab, Imdevimab, Bamlanivimab, and Etesevimab). We diluted antibodies in cell culture media with 2-fold serial dilutions, with the final concentration of 10 µg/ml to 0.02 µg/ml in triplicate. Viruses were diluted in cell culture media and added to the diluted antibody series in wells of 96-well U-bottom plates to an adjusted titer of 100 plaque-forming units. Plates were incubated at 37°C for 30 min. Culture supernatant was aspirated from the Vero E6/TMPRSS2 cells plated in tissue culture 96-plates and replaced with the mixtures of serially diluted antibody and virus mixture (100 µl/well) followed by incubation at 37°C with 5 per cent CO_2_ for 3 days.

The inhibitor concentration that prevented 99 per cent cell death (IC99 value) at the given concentration (as determined by cytopathic effects visualized by a bright-field microscope) defines antibody potency.

### SARS-CoV-2 sequencing

We performed sequencing on a nasopharyngeal or nasal swab sample that was leftover after twelve of the immunocompromised individual’s PCR tests. All viral RNA extractions over the course of this study took place on the Maxwell RSC Viral Total Nucleic Acid Purification Kit (Promega, USA), used according to the manufacturer’s instructions.

To generate sequence data, we first sequenced viral cDNA libraries on an Oxford Nanopore (ONT) minION with the ARTIC v3 amplicon approach (https://www.protocols.io/view/covid-19-artic-v3-illumina-library-construction-an-bibtkann), but the previously documented amplicon dropout near the beginning of the Spike gene occurred in all of our samples. To improve the breadth of coverage across the whole of the Spike gene, we re-sequenced ten of the samples on an ONT MinION using the MIDNIGHT v1 protocol (https://www.protocols.io/view/sars-cov2-genome-sequencing-protocol-1200bp-amplic-bwyp-pfvn) (Freed et al. 2020). To examine minor variant frequencies with the maximum breadth of coverage, we later used the ARTIC v4.1 protocol to sequence overlapping ten samples on an Illumina MiSeq (https://www.protocols.io/view/sars-cov-2-sequencing-on-illumina-miseq-using-arti-bfefjjbn). Reaction volumes for and all other details for our ARTIC v3, ARTIC v4.1, and MIDNIGHT v1 procedures are available at https://go.wisc.edu/89wtu8.

### SARS-CoV-2 sequence analysis

#### Processing raw sequence data for consensus sequence analyses

The majority of analyses described here use consensus sequences, which we assembled for each sample using a pipeline customized to run remotely with compute resources from the University of Wisconsin-Madison Center For High Throughput Computing (https://chtc.cs.wisc.edu/). For each consensus sequence, ONT MinION reads first go through ONT’s live basecalling, which automatically fails reads with low-quality bases in real time. The custom pipeline then maps raw reads with a length between 400 and 700 bases to MN908947.3 (Wuhan-1 SARS-CoV-2) using the ‘-a -x map-ont’ preset in minimap2 (Li 2018). For all positions with greater than twenty reads depth-of-coverage, the pipeline then calls the bases occurring in 50 per cent or more of the reads as the consensus bases. Finally, the pipeline formats consensus sequences and metadata for upload to public data repositories including GenBank (see Supplementary Table S1). In the workflow we designed to visualize data in this study, we identify nucleotide variants in each consensus sequence using iVar, which produces tab-delimited outputs that are straightforward to plot in R (Grubaugh et al. 2019).

To visualize amplicon dropouts in these consensus sequences, we returned to the ONT MIDNIGHT reads in FASTQ format, which were originally used to generate consensus sequences. First, we mapped the reads to MN908947.3 using minimap2’s ‘map-ont’ preset, clipped reads down to MIDNIGHT amplicon pile-ups using SAMtools, and then used covtobed to produce BED-format tables of regions where read depth-of-coverage was below 20 (Li 2018; Danecek et al. 2021; Birolo and Telatin 2020). Finally, we used an R script to plot mutations in the consensus sequences through time, along with dropouts identified in the raw read data.

#### Processing Illumina reads for variant frequency analyses

While our sequence analysis pipeline largely uses MIDNIGHT primer set ONT data, sequences from this platform come with a relatively high error rate. This error rate can obscure low-frequency variants when examining intrahost variant frequencies. To overcome this obstacle, we sequenced leftover samples from ten time points on an Illumina MiSeq as described above. We then quality-controlled and called variants from our Illumina reads using version 2.5 of the nf-core/viralrecon pipeline, an open source NextFlow pipeline designed to assemble and call low-frequency, intrahost variants from virus samples (Patel et al. 2022). From there, variants that emerged after Bamlanivimab treatment were plotted using an R script. All code, data, and replication instructions for our Illumina data processing and plotting are available at https://github.com/dholab/prolonged-infection-suppfig1.

#### Genetic distance analysis

To examine the amount of mutations the patient’s virus accumulated over the course of the infection, and compare that accumulation with SARS-CoV-2 globally, we randomly subsampled approximately 5,000 SARS-CoV-2 sequences from GenBank. We then aligned the GenBank consensus sequences to MN908947.3 using minimap2 (Li 2018), called variants from all sequence alignments using the BBTools ‘callvariants.sh’ script (BBMap 2021), and then used R to count the number of mutations from MN908947.3 and plot (see https://github.com/dholab/prolonged-infection-suppfig2 for all code, data, and replication instructions for this analysis).

## Supplementary Material

veac104_Supp

## Data Availability

All scripts, consensus sequence data, and sequencing read data used in the writing of the main text of this manuscript can be found at https://github.com/dholab/E484T-visualizations. Workflows and data used for Supplementary Figs S1 and S2 are available at https://github.com/dholab/pro-longed-infection-suppfig1 and https://github.com/dholab/prolonged-infection-suppfig2, respectively. Raw read data, concatenated for each sequencing time point, are also available in National Center for Biotechnology Information (NCBI) BioProject #PRJNA836936. Additionally, all scripts, input and output files, intermediate data files, run statistics, and other associated data are available on our data availability portal at https://go.wisc.edu/89wtu8.
