# Supplementary material for: Evolution of a globally unique SARS-CoV-2 Spike E484T monoclonal antibody escape mutation in a persistently infected, immunocompromised individual
Source: Virus Evol. 2022 Nov 5;9(2):veac104. doi: 10.1093/ve/veac104 (PMC10491860; doi:10.1093/ve/veac104)
Supplement: veac104_Supp [file veac104_supp.zip › suppl_data/supplemental_materials_titles_captions.docx]

# Supplemental Figures

## Supplemental Figure 1.

Intrahost single-nucleotide variants (iSNVs) that reach ≥50% (consensus) frequency after the March 2021 Bamlanivimab monoclonal antibody treatment. One of the iSNVs that reached consensus frequency at post-diagnosis day 297, ORF1ab V4102I, was present at a prior timepoint, albeit at lower frequencies among sequencing reads (see Supplemental Tables 2 and 4).

## Supplemental Figure 2.

Root-to-tip analysis of chronic infection virus compared to a global subsample of 5,000 GenBank SARS-CoV-2 uploads. All points represent a sum of genetic differences between the sample consensus sequence and Wuhan-1 (GenBank: MN908947.3). Global SARS-CoV-2 samples show genetic distances that increase at a similar rate to this chronic infection virus. A pronounced increase in mutations occurs after the Bamlanivimab monoclonal antibody treatment on day 198.

## Supplemental Figure 3.

The number of intrahost single-nucleotide variants observed at each amino acid residue within each varying gene. No residues showed elevated iSNV diversity in our sequencing timepoints, including Spike residue 484.

# Supplemental Tables

## Supplemental Table 1

Due to the collaborative nature of this project, virus in the immunocompromised individual’s nasopharyngeal (NP) swab samples was quantified and sequenced using different platforms throughout the infection. All samples were sequenced with either the MIDNIGHT protocol on an Oxford Nanopore MinION, the ARTIC protocol on an Illumina MiSeq, or both.

## Supplemental Table 2

25 Intrahost Single-Nucleotide Variants (iSNVs) that had risen above 0.5 frequency by post-diagnosis day 297, with iSNV frequency among sequencing reads and depth-of-coverage compared between two library preparation methods. The nucleotide substitution that causes E484A, A-23013-C, is also included. Note that while G-23012-A causes Spike E484K in isolation, as it is listed in this table, the substitution caused Spike E484T in the patient’s virus as a second step from Spike E484A.

## Supplemental Table 3

All unique single nucleotide variants that occurred in greater than or equal to 50% of the reads in at least one of our twelve timepoints. These consensus mutations often occurred at multiple timepoints, occasionally falling to a frequency lower than 50% (see Supplemental Table 4).

## Supplemental Table 4

All observed Intrahost Single-Nucleotide Variants (iSNVs) at all time points, together with Illumina sequencing read depths and frequencies of each variant among those reads. Visualization of these reads through time is available at <https://github.com/dholab/prolonged-infection-suppfig1/tree/main/3_plot_data/results/visuals>
