# Supplementary material for: Evolution of a globally unique SARS-CoV-2 Spike E484T monoclonal antibody escape mutation in a persistently infected, immunocompromised individual
Source: Virus Evol. 2022 Nov 5;9(2):veac104. doi: 10.1093/ve/veac104 (PMC10491860; doi:10.1093/ve/veac104)
Supplement: veac104_Supp [file veac104_supp.zip › suppl_data/unabbreviated_case_description.docx]

### Unabbreviated Case Description

The 57-year-old patient described in this case suffers from a combination of immune system disorders and is on appropriate medical prophylaxis. The patient’s medical history is notable for common variable immunodeficiency (CVID) with recurrent lower respiratory tract infections, and managed with ongoing monthly IVIG infusions since 2015. Also in the patient’s history is Evans syndrome and associated autoimmune cytopenias, as well as extranodal mucosa-associated lymphoid tissue (MALT) lymphoma of the lung. The patient’s lymphoma was previously treated with bendamustine and rituximab, though a number of complications arose, including prolonged neutrophil recovery, baseline mild bronchiectasis, hemothorax, and mild scattered subpleural scarring.

This case began when the patient presented to their primary care provider on 5 September 2020, reporting sinus congestion and pleurisy, though notably without fevers, chills, loss of taste or smell, or shortness of breath. The patient was treated with augmentin and concurrently gave a nasopharyngeal swab sample to be PCR-tested for SARS-CoV-2 infection. That swab sample soon came back positive, with a cycle threshold value of 26.8, making 5 September 2020 the date of diagnosis and what we refer to here as post-diagnosis day 0.

After an initial clinical recovery, the patient developed recurrent daily fevers, shortness of breath, dyspnea on exertion, and fatigue by day 52. Chest x-ray showed right lower lobe opacity. The patient was treated with azithromycin for community-acquired pneumonia, and subsequently doxycycline for persistent fevers. Nonetheless, symptoms continued through day 66, at which time they presented to an outside hospital emergency department with persistent fevers and right lower lobe opacities. The patient was admitted to an outside hospital for further workup and management on day 66.

Hospital admission lasted from post-diagnosis day 66 to 70, during which they received intravenous ceftriaxone and repeat nasal pharyngeal SARS-CoV-2 PCR-testing (positive on days 67 and 68) with negative SARS-CoV-2 IgG. On day 66, a chest CT showed multifocal ground glass opacities, right greater than left, consistent with an organizing pneumonia pattern of injury related to COVID-19. The patient also underwent a full infectious disease workup, including bronchoscopy, on day 68, but there were no positive results to suggest an infection other than SARS-CoV-2. Transbronchial biopsies taken during bronchoscopy also revealed no evidence of infection, but were consistent with diffuse alveolar damage. As such, the patient’s antibiotics were discontinued, and they were discharged home with no supplemental oxygen at that time. Their oxygen saturation remained in the mid-90s mmHg, though with dips into the high 80’s during activity. Other than hypoxia and recurrent fevers, other symptoms had resolved by discharge at day 70.

Given the patient’s symptoms, imaging results, an erythrocyte sedimentation rate greater than 140, and an absence of other identifiable infectious etiology, the patient was started on a 10-day course of outpatient dexamethasone on day 74, which was followed by improvement in fevers. Another broad peripheral fungal workup returned negative. At that time, the patient began developing progressive hypoxia, and was soon given 4 liters of supplemental oxygen via nasal cannula for use during activity and sleep. Notably, on day 87, a nasopharyngeal swab specimen from a member of the patient’s household tested positive for SARS-CoV-2 infection, though no primary sample from this test was available for sequencing. That same day, patient was restarted on a 10-day regimen dexamethasone, with 4 mg for 5 days followed by 2 mg for 5 days.

On day 103, the patient underwent video-assisted thoracic surgery (VATS) with wedge biopsies of the upper and lower left lung lobes, which showed further evidence of organizing pneumonia, diffuse alveolar damage, and chronic lung fibrosis. Following this procedure, the patient’s respiratory status worsened, with blood oxygen desaturation into the 70s. In response, the patient was placed on bilevel positive airway pressure (BiPAP), which improved oxygenation. On day 107, the patient was started on cefepime, vancomycin, and anidulafungin due to concern for superimposed bacterial or fungal infection.

About a week later, on day 113, the patient became progressively hypoxemic and was admitted to the ICU for intubation. Chest x-ray in the ICU showed multifocal and peripherally-prominent airspace opacities and bibasilar atelectasis. The initial diagnosis was recorded as: “acute on chronic hypoxic respiratory failure, with evidence of COVID-19 pneumonia, and concerning progression of post-viral organizing bacteria pneumonia, other opportunistic infection, or underlying interstitial lung disease.” On day 116, the patient was able to be extubated, but was still unable to wean off high flow nasal cannula. On day 124, the patient again tested PCR-positive for SARS-CoV-2.

Following the positive SARS-CoV-2 test result on day 124, the patient received two courses of inpatient remdesivir and two units of convalescent plasma. The patient was also maintained on an aggressive steroid regimen of stress dose methylprednisolone, followed by dexamethasone and a prolonged prednisone taper. At this time, the patient’s physician team requested compassionate use of monoclonal antibodies from Eli Lilly and Regeneron, but both requests were rejected. Another positive PCR test result came in on day 132. The following day, the patient received their monthly IVIG infusion. On day 137, the patient was discharged, but maintained on a steroid regimen and 2 liters of supplemental oxygen via nasal cannula. Upon an outpatient follow-up visit on day 159, the patient’s nasopharyngeal swab was again PCR-positive for SARS-CoV-2. Repeat chest imaging showed ongoing organizing pneumonia, with reduced ground-glass opacities and residual lung fibrosis. Pulmonary function tests showed a severe restrictive ventilatory defect compared to the normal pulmonary function from before SARS-CoV-2 infection.

The patient continued to experience irregular fevers, myalgias, and fatigue over the following weeks, and eventually presented to an outpatient pulmonary specialist on day 186. Prior to this visit, the patient had been receiving ongoing monthly IVIG and intermittent prednisone bursts and taper, but symptoms persisted nonetheless. The patient’s physicians prescribed celecoxib for fever and submitted another request for compassionate use of a monoclonal antibody therapy from Eli Lilly. This request was approved, and the patient received 700mg of intravenous Bamlanivumab on day 198, the same day as another PCR-positive nasopharyngeal swab. Notably, this treatment was prior to the FDA’s revocation of the Emergency Use Authorization for this monotherapy. On day 255, the patient reported worsening shortness of breath and low-grade fever, and chest CT scans again showed organizing pneumonia. Nasopharyngeal swabs came back positive on day 297 and 333, though without any major changes in symptoms and pulmonary function, which waxed and waned over the following months.

On day 382, the patient was admitted to a second facility for a second opinion on their worsening dyspnea and cough. Nasopharyngeal swab (NP) was positive by PCR for SARS-CoV-2, with a cycle threshold of 24.20. Repeat left lung imaging demonstrated improvement in the lung’s fibrotic opacities, but discovered a new cavitary nodule in the lower left lung. Semi-quantitative detection of total antibodies against SARS-CoV-2 Spike protein (Roche Elecsys Anti-SARS-CoV2 S assay) resulted in titers of 102 U/mL. The patient subsequently received three infusions of high-titer convalescent plasma therapy as well as 5 days of IV remdesivir. Bronchoscopy with bronchoalveolar lavage (BAL) was performed, but the BAL sample was negative for SARS-CoV-2 infection, as were cultures for bacterial, mycobacterial and fungal infections. SARS-CoV-2 testing from the BAL sample resulted negative on day 387. Still, the patient’s nasopharyngeal swabs remained PCR-positive for SARS-CoV-2 infection on days 388 and 405.

On day 415, the patient was started on 3 days of outpatient VaxPlasma, a high-titer post-infection vaccine-derived donor plasma under the FDA emergency IND protocol. The infusion increased the patient’s anti-spike protein antibody serum titers from 105 to >250 U/mL. Still, on days 415, 417, 432, and 433, nasopharyngeal swabs from the patient remained PCR-positive. Hypoxemia, requiring two to three liters per minute of supplemental oxygen, and dyspnea at rest persisted during those timepoints. On day 475, a transbronchial biopsy from the patient’s upper left lung lobe tested PCR-positive for SARS-CoV-2 RNA. The patient received their monthly IVIG infusion on day 478, but a week later, on December 31st, 2021, post-diagnosis day 482, the patient’s nasopharyngeal swab again tested PCR-positive. The patient subsequently received another five-day course of IV remdesivir and another three infusions of VaxPlasma. Before the VaxPlasma infusion, the patient’s anti-spike antibody serum titer was 687 U/mL, whereas afterward it had risen to 831 U/mL. Nonetheless, another nasopharyngeal swab tested positive on day 486, though with the highest Ct of their infection (Supplemental Table 1).

Eventually, on post-diagnosis day 525, the patient tested PCR-negative for SARS-CoV-2 infection. At four and eight months after this initial negative, the patient again tested PCR-negative, though they still suffer post-COVID lung damage and loss of taste and smell.
