# Supplementary figures and images for: Evolution of a globally unique SARS-CoV-2 Spike E484T monoclonal antibody escape mutation in a persistently infected, immunocompromised individual
Source: Virus Evol. 2022 Nov 5;9(2):veac104. doi: 10.1093/ve/veac104 (PMC10491860; doi:10.1093/ve/veac104)

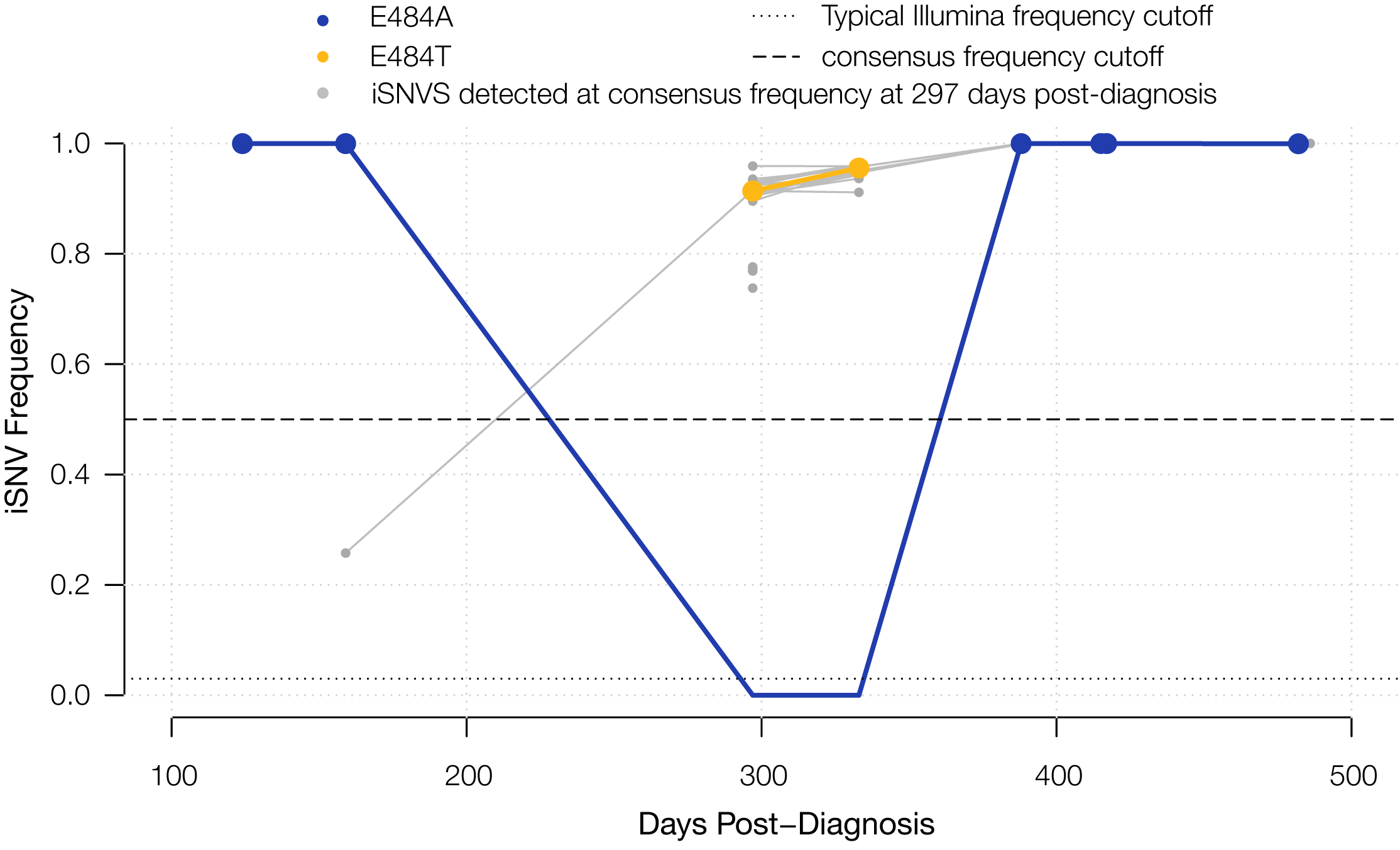

Supplement: veac104_Supp [file veac104_supp.zip › figsupp1_isnv_frequencies_lzw.tif]

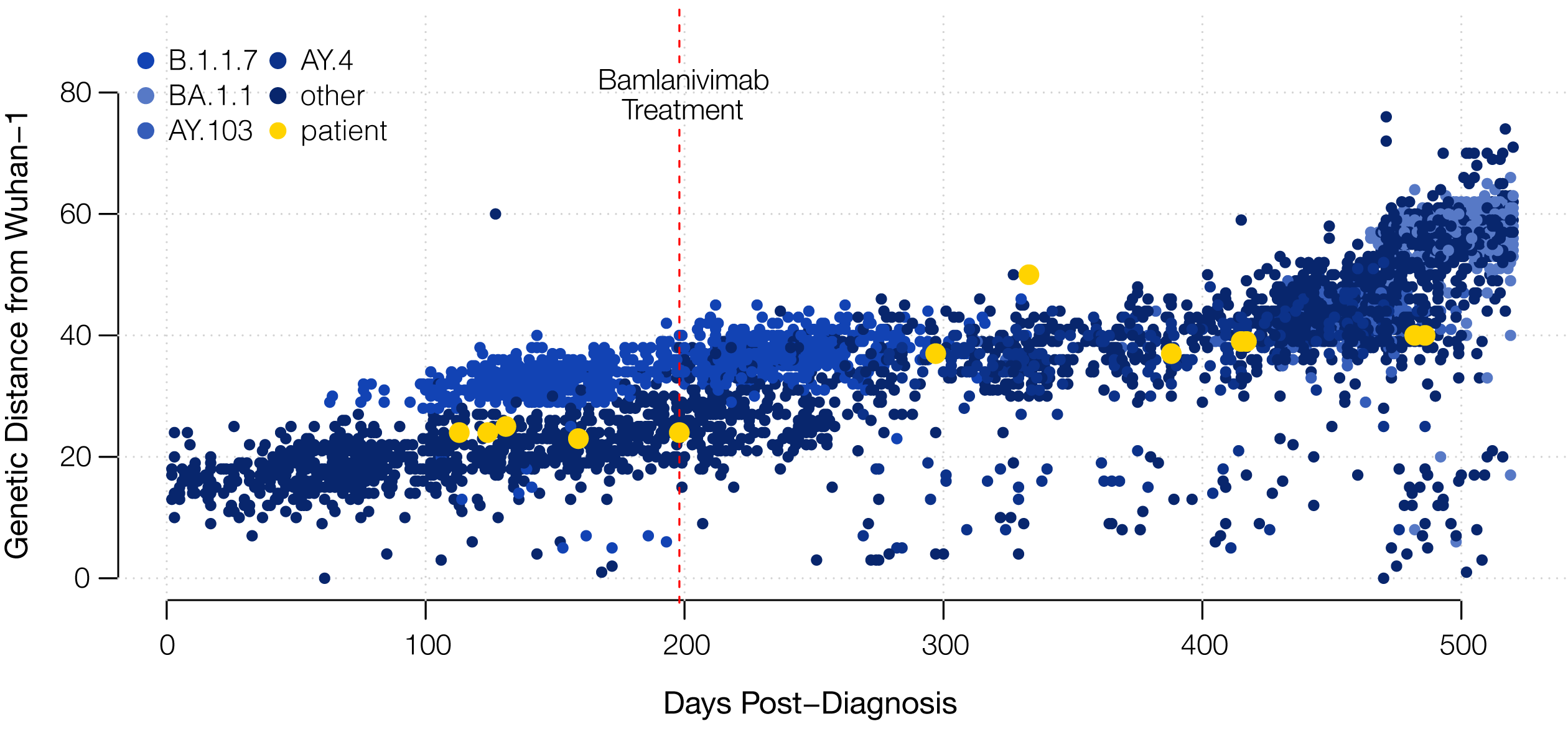

Supplement: veac104_Supp [file veac104_supp.zip › figsupp2_global_roottotip_plot_lzw.tif]

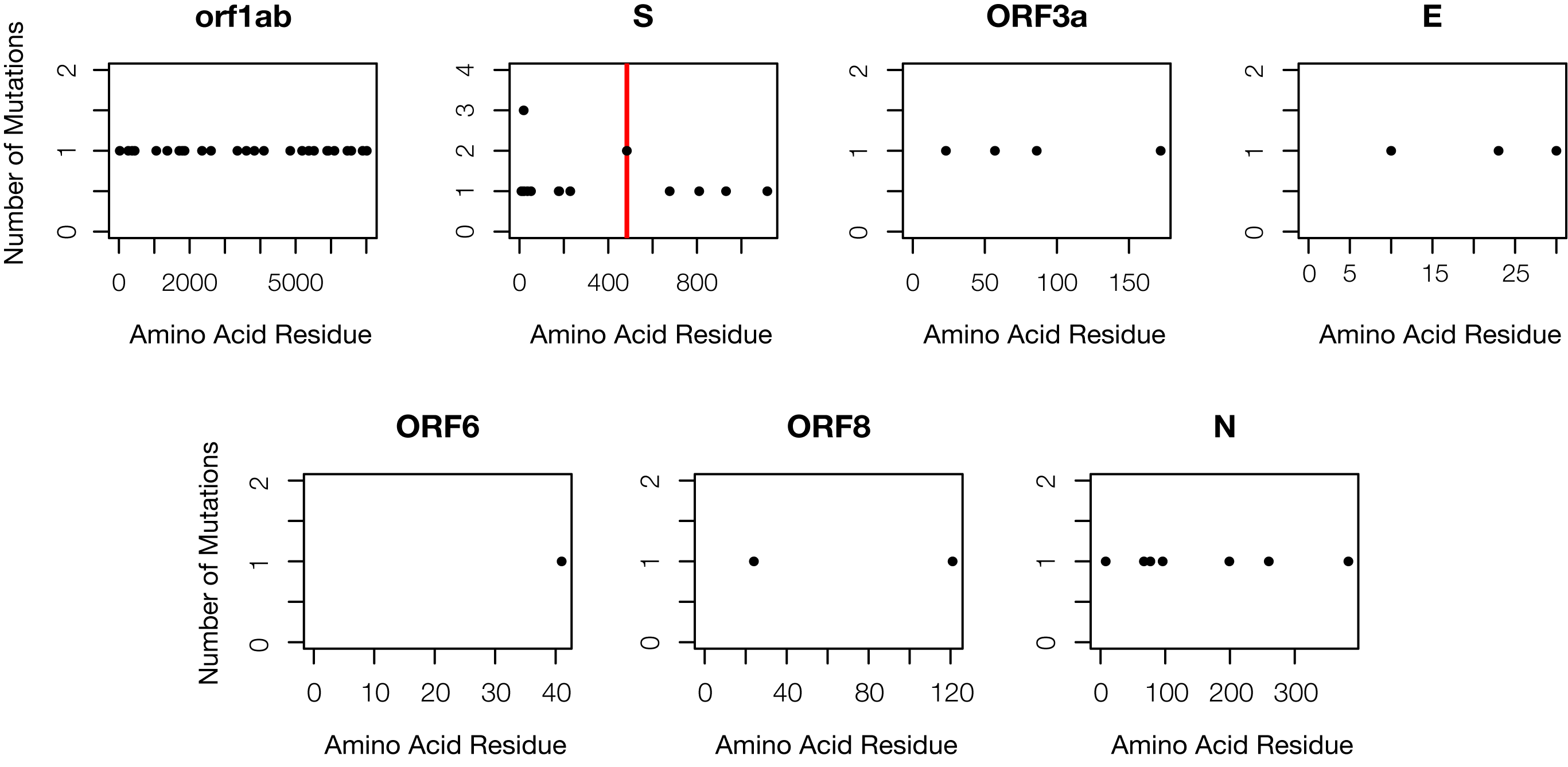

Supplement: veac104_Supp [file veac104_supp.zip › figsupp3_outlier_epitopes_lzw.tif]
